# The Effect of Age on 1‐Year Readmissions in Atrial Fibrillation Patients: Trends and Insights From a Conflict‐Stricken Country

**DOI:** 10.1002/clc.70186

**Published:** 2025-07-26

**Authors:** Ibrahim Antoun, Alkassem Alkhayer, Aref Jalal Eldin, Alamer Alkhayer, Riyaz Somani, G. André Ng, Mustafa Zakkar

**Affiliations:** ^1^ Department of Cardiovascular Sciences University of Leicester Leicester UK; ^2^ Faculty of Medicine University of Aleppo Aleppo Syria; ^3^ Faculty of Medicine University of Tishreen's Hospital Latakia Syria; ^4^ Department of Cardiology University Hospitals of Leicester NHS Trust, Glenfield Hospital Leicester UK; ^5^ Department of Cardiac Surgery University Hospitals of Leicester NHS Trust, Glenfield Hospital Leicester UK; ^6^ Faculty of Medicine University of Damascus Damascus Syria

**Keywords:** atrial fibrillation, conflict, outcomes, readmission, Syria

## Abstract

**Background:**

Atrial fibrillation (AF) is a leading cause of cardiovascular morbidity and hospitalization worldwide. However, limited data exist on AF readmissions in low‐resource and conflict‐affected settings. This study investigates the impact of age on 1‐year readmission rates among AF patients in a Syrian tertiary hospital.

**Methods:**

This retrospective observational cohort study was conducted at a tertiary Syrian center between June/2021–November/2023. Patients admitted with primary AF were included, while those with secondary AF or missing demographic data were excluded. Patients were stratified into three age groups: 18–50 years (Group 1), 51–70 years (Group 2), and > 70 years (Group 3). The primary outcome was all‐cause and cardiovascular‐related 1‐year readmissions, with secondary outcomes including readmission frequencies.

**Results:**

A total of 657 AF patients were included, with a median age of 60 320 (52%) were males. One‐year readmission occurred in 64% of patients, with AF being the most common cause (75%). Group 1 had the highest smoking rates (70%). Group 3 had the highest rates of ischemic heart disease (47%), congestive cardiac failure (CCF) (35%), chronic kidney disease (15%, *p* < 0.001) and chronic liver disease (20). Older age was significantly associated with increased readmissions (87% in Group 3 vs. 62% in Group 2 and 49% in Group 1, *p* < 0.001). Frequent readmissions were more prevalent in Group 3 (≥ 3 admissions: 46%).

**Conclusion:**

Older AF patients in a conflict‐affected setting experience significantly higher readmission rates. Addressing healthcare resource limitations and optimizing AF management strategies are crucial to improving outcomes in resource‐limited settings.

## Graphical abstract

Older age significantly increases 1‐year readmission risk among atrial fibrillation patients in a conflict‐affected Syrian cohort. Atrial fibrillation (AF) recurrence was the leading cause. Findings underscore the need for improved follow‐up and resource allocation to manage AF in fragile healthcare systems.

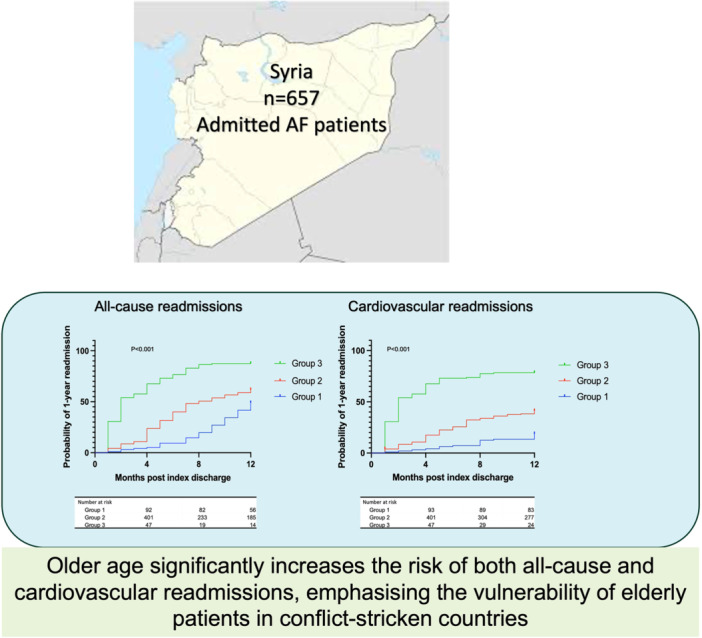

## 1 Introduction

Atrial fibrillation (AF) is the most common type of arrhythmia worldwide, and its prevalence in middle—to low‐income countries is likely underestimated [1]. Although AF in the developed world is well studied, there is little data on AF management patterns in the Middle East [2].

One of these countries, Syria, has been suffering from a conflict for the past 14 years. This has left the country deprived of healthcare funding and resources, which was exacerbated during the COVID‐19 pandemic and cholera outbreak [3, 4]. Less than half the hospitals operate at full capacity, and more than half of the healthcare workers are forced to flee the country due to escalating conflict [5]. AF management in Syrian hospitals during the current economic and political turmoil is unclear, with a paucity of published inpatient figures and outcomes originating from Syrian healthcare [6, 7, 8, 9, 10, 11, 12, 13, 14]. A real‐world depiction of the current AF care in the context of resource limitations can help manage and allocate resources by recognizing remediable deficiencies and identifying practical and reasonable solutions that can be enforced [6]. Although advances in AF management have enhanced symptom control, readmission rates continue to increase and have been one of the main sources of AF‐related financial constraints on healthcare economies [15]. Particularly for Syria, following up on patients after initial admissions related to AF is challenging due to limited healthcare resources and damaged infrastructure [5]. The role of age in increasing readmission rates was studied extensively in developed countries [15, 16, 17, 18, 19] but not in developing and conflict‐stricken countries. This study aims to assess the contribution of age to readmissions in this cohort, providing insights into the burden of AF‐related hospitalizations among older individuals in resource‐limited settings.

## 2 Methods

### 2.1 Design and Data Collection

This is a retrospective single‐center observational cohort study conducted at Tishreen's University Hospital in Latakia, Syria, from June 2021 to November 2023. The hospital is a large government‐operated public institute associated with Tishreen University, and it serves as the primary healthcare center for the city and the surrounding areas. The hospital has 860 beds and provides free healthcare to Syrian citizens. The hospital cares for approximately 50 000–60 000 inpatients annually, with more outpatients seeking care in various departments. The study included patients over 18 years old who were treated for AF as the primary diagnosis during index admission. Patients with secondary AF and missing data for sex and age were excluded. Patients were followed for 1 year after discharge from their initial admission to track readmissions. Data sources for the study included hospital paper and electronic records. The medical or cardiology consultant determined the causes of the index admission and readmissions. The patients were split into three groups based on age. Group 1 includes patients aged 18–50 years; Group 2 includes patients aged 51–70, and Group 3 is aged > 70 years. The research reported in this article adhered to the Declaration of Helsinki. The study was conducted as part of an audit approved by the hospital board and involved prospective analysis of retrospectively collected anonymised data. Therefore, the ethical committee of Tishreen's University Hospital waived the need for consent. The study was reported according to the STROBE statement [20].

### 2.2 Outcomes

Our study's primary outcome was aetiologies of readmissions and 1 year of all‐cause and cardiovascular readmissions in different age groups. Secondary outcomes include frequencies of readmissions across the different age groups.

### 2.3 Statistical Analysis

Continuous variables are expressed as medians and interquartile ranges (IQR), while categorical variables are expressed as counts and percentages (%). Pearson's χ 2 or Fisher's exact test was used to compare categorical variables between groups, while the Student's *t*‐test was used to compare continuous variables.

Survival analysis was used to investigate the relationship between age and the probability of readmission within 1 year. We hypothesized that increased age would positively affect the 1‐year readmission probability. Statistically significant comorbidities in the univariate analysis were added to the multivariable analysis. A 2‐sided *p*‐value < 0.05 was considered statistically significant. Statistical analysis was performed using GraphPad Prism V10.3 for Mac (San Diego, California, USA).

## 3 Results

### 3.1 Baseline Characteristics

Over the study period, an estimated 150 000 patients attended the emergency department, of which 657 had an index admission with primary AF. The median age was 60, and 320 (52%) were males. Among the patients, 422 (64%) had an unplanned readmission within 1 year after index discharge. Table 1 demonstrates demographics. Older age was strongly associated with higher readmission risk and a greater burden of comorbidities. Group 1 had the lowest cardiovascular disease prevalence but the highest smoking rates (70%, *p* < 0.001). In contrast, Group 2 showed a significant increase in ischemic heart disease (IHD) (25%, *p* < 0.001) and chronic kidney failure (9%, *p* < 0.001). Group 3 had the highest rates of ischemic heart disease (47%, *p* < 0.001) and congestive cardiac failure (CCF) (35%, *p* < 0.001). They also had significantly higher rates of chronic kidney disease (15%, *p* < 0.001) and chronic liver disease (20%, *p* < 0.001). The CHA_2_DS_2_VA score increased with age, reaching a median of 4 in Group 3 (*p* < 0.001), indicating a higher stroke risk. These findings highlight age as a key determinant of readmission, with significant associations between older age, comorbidities, and increased healthcare burden. All patients in the study had rate control, while 150 patients (23%) had rhythm control, with amiodarone being the only available rhythm control method. There was no statistical difference between age groups regarding perusing a rhythm control strategy.

**TABLE 1:** Baseline characteristics of patients with 6‐months readmission versus no readmission after index admission with acute atrial fibrillation.

	Overall (*n* = 657)	Age group 1 (18–50) (*n* = 96)	Age group 2 (51–69) (*n* = 450)	Age group 3 (> 70) (*n* = 111)	*p*‐value
Demographics, n (%) or median (IQR)					
Age (years)	60 (54–67)	45 (39–49)	60 (55–65)	76 (74–80)	< 0.001
Male	340 (52%)	102 (45%)	148 (63%)	90 (81%)	< 0.001
Cardiovascular comorbidities, n (%) or median (IQR)					
Current smoking	397 (60%)	67 (70%)	243 (54%)	87 (78%)	< 0.001
Hypertension	221 (34%)	25 (26%)	151 (34%)	45 (41%)	0.08
Ischemic heart disease	171 (26%)	7 (7%)	112 (25%)	52 (47%)	< 0.001
Diabetes mellitus	145 (22%)	26 (27%)	91 (20%)	28 (25%)	0.23
Cerebrovascular disease	123 (19%)	12 (13%)	83 (18%)	28 (25%)	0.06
Congestive heart failure	123 (19%)	14 (15%)	74 (16%)	39 (35%)	< 0.001
Thyroid disease	22 (3%)	6 (6%)	12 (3%)	4 (4%)	0.2
CHA_2_DS_2_VA score	2 (1‐3)	2 (1‐2)	2 (1‐3)	4 (3‐4)	< 0.001
Other comorbidities, n (%)					
Active malignancy	44 (7%)	0 (0%)	28 (6%)	16 (14%)	0.01
Chronic liver failure	50 (8%)	0 (0%)	28 (6%)	22 (20%)	< 0.001
Chronic lung disease	72 (11%)	3 (3%)	56 (12%)	13 (12%)	0.03
Chronic kidney failure	58 (9%)	0 (0%)	41 (9%)	17 (15%)	< 0.001
Treatments, n (%)					
Rate control	657 (100%)	96 (100%)	450 (100%)	111 (100%)	0.99
Rhythm control^a^	150 (23%)	24 (25%)	99 (22%)	27 (24%)	0.77

Abbreviation: IQR, interquartile ranges.

^a^ Amiodarone was the only rhythm control drug available.

### 3.2 Aetiologies of 1‐Year Readmissions

**FIGURE 1:**
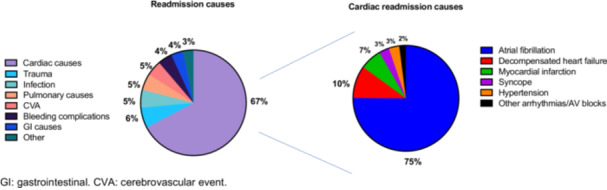
Aetiologies of 1‐year readmissions after index admission for atrial fibrillation. GI: gastrointestinal. CVA: cerebrovascular event.

Aetiologies of 1‐year readmission are demonstrated in Figure 1. Cardiac conditions were the most common cause of 1‐year readmission (67%). Of cardiac conditions, the most common condition was AF (75%), followed by decompensated CCF (10%) and myocardial infarction (7%). The most common noncardiac causes of readmission were trauma (6%), infection, pulmonary causes and cerebrovascular events at 5% each. Among AF‐related readmissions, 80% were due to recurrence of symptoms requiring rate or rhythm control. Approximately 12% were attributed to treatment‐related complications, predominantly bleeding or thromboembolic events linked to anticoagulation. The remaining 8% were related to inadequate post‐discharge care, including poor medication adherence and limited outpatient access.

### 3.3 Readmissions in the Age Groups

**FIGURE 2:**
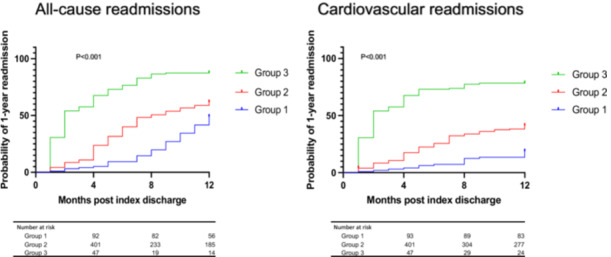
Cumulative incidence curves navigating 12 months of readmission in different age groups in the study cohort.

Cumulative incidence curves of all‐cause and cardiovascular readmissions in all age groups are demonstrated in Figure 2. We identified that group 3 were associated with an increased risk of all‐cause readmission (87%) compared to group 2 (62%) and group 1 (49%) at 1 year (*p* ≤ 0.001). Similarly, group 3 patients were associated with an increased risk of cardiovascular readmissions (78%) compared to group 2 (41%) and group 1 (19%) at 1 year (*p* ≤ 0.001).

### 3.4 Trends and Frequencies in Readmissions

The readmission rates for patients in group 1 were 16%, 9%, and 6%, respectively. For those in group 2, the rates were the same: 21%, 22%, and 26%. Group 3 patients were readmitted once, twice, or more, respectively, at 26%, 39%, and 46%.

## 4 Discussion

This study is the first to study the effect of age on 1‐year readmission following index admission for primary AF in the Middle East and Syria. It highlights several novel findings specific to our Syrian population. First, 64% of Syrian patients were readmitted within 1 year of their index admission, with AF being the most common cause. Second, older age was associated with increased odds of all‐cause and cardiovascular readmissions. Lastly, frequent admissions were common in the older age group.

As AF has considerable implications on economies worldwide [21], recent studies have focused on many aspects of AF, including hospitalization, quality of life, treatment patterns, and readmission rates [6, 8, 16]. Our 1‐year readmission rate of 64% far exceeds the rates reported in the developed world of 12.5% [22]. Furthermore, our 1‐year readmission rate exceeds the 2‐year readmission rate in Australia of 44% [23]. There was no data from low‐to‐middle‐income countries to compare. Our higher readmission rate can be explained by the ongoing conflict in Syria since 2011, which has primarily affected health infrastructure and caused an increased turnover of skilled staff and inadequate numbers of nurses and allied health professionals [24]. As only half the primary health care centers and hospitals are fully functional in Syria [24], managing risk factors and following up with patients presenting to hospitals with primary AF after discharge is challenging.

Also, access to medications during conflict has been challenging and should be addressed by international health organizations [25]. Patient engagement in high‐risk behaviors, mainly smoking, is correlated with unfavorable AF clinical outcomes [26]. For example, a recent Syrian study during the conflict demonstrated a smoking rate of 38% in 978 participants who had a mean age of 25 years, which was described as worrying [27]. Therefore, patient education and tackling these AF risk factors are essential to reducing the readmission burden and optimizing outcomes in resource‐depleted communities such as Syria.

Although there was no similar data before the conflict, supporting the Syrian health care system, including primary care, would help reduce the 1‐year readmission rate in Latakia and nationwide. Our cohort's most common readmission causes were cardiac, with AF being the most common. This was in keeping with previous literature [15, 16]. It is common for AF patients to be readmitted with the same cause. For instance, in the Framingham study, only 10% of AF patients were classified as experiencing no recurrence in the community during the 2‐year follow‐up period [28]. This can be attributed to many AF patients having underlying conditions that often go unrecognized, alongside various coexisting comorbidities that may trigger AF. This is proposed to commonly happen in conflict situations where access to healthcare and resources is limited. This situation likely causes early subsequent frequent readmissions, as proven in our cohort, with 73% of patients having readmissions within the year following index admission. This can also be explained by the disparity in discharge planning, access to health care, lack of care coordination, insufficient continuity of care and limited availability of specialized cardiac services. Although data on AF‐specific readmissions in other conflict‐affected regions are limited, studies from Iraq, Afghanistan, and sub‐Saharan Africa describe similar healthcare challenges, including high cardiovascular morbidity, disrupted access to long‐term care, and inconsistent availability of essential medications [29, 30, 31]. These parallels suggest that the high readmission rates observed in our Syrian cohort may reflect broader chronic disease management failure patterns in fragile health systems. Our findings thus offer relevant insights into AF management and outcomes in Syria and other regions affected by prolonged conflict and healthcare disruption.

Our study demonstrates a clear association between older age and increased 1‐year readmission rates in AF patients, a finding that aligns with existing literature from developed healthcare settings [15, 32]. Previous studies have shown that advancing age is an independent predictor of hospital readmission due to the higher burden of comorbidities, increased frailty, and a limited physiological reserve, all of which contribute to poor post‐discharge outcomes [33, 34]. The elderly population is particularly vulnerable to complications such as heart failure, cerebrovascular disease, and chronic kidney disease, conditions that frequently lead to hospital readmissions [35]. In our cohort, patients over 70 years of age exhibited the highest rates of cardiovascular readmissions (78%), largely due to recurrent AF, decompensated CCF and myocardial infarction.

The complexity of AF management also influences the increased readmission risk in older patients in this age group. Polypharmacy, limited adherence to medications, and higher bleeding risks with anticoagulation therapy further complicate disease control, increasing the likelihood of hospitalization [36]. Additionally, older adults are more likely to have undiagnosed or poorly managed AF risk factors such as hypertension and diabetes, which exacerbate disease progression [37]. In a resource‐limited setting such as Syria, where healthcare infrastructure is weakened by conflict, the challenges of managing elderly AF patients are amplified due to restricted follow‐up, medication shortages, and inadequate access to specialist care [38].

Beyond the initial readmission, our findings indicate that recurrent hospitalizations are common in elderly AF patients, with a significant proportion experiencing multiple readmissions within 1 year. Specifically, 46% of patients over 70 were readmitted at least three times within the follow‐up period. This aligns with prior research demonstrating that AF is a chronic and relapsing condition, often leading to multiple hospital visits due to persistent or worsening symptoms [39]. The Framingham Heart Study reported that recurrent AF episodes and their associated complications lead to frequent rehospitalisation, with older adults being at the highest risk [40].

Frequent hospitalizations impose a significant financial and logistical burden on healthcare systems, particularly in low‐resource settings. Studies from high‐income countries have highlighted that AF‐related admissions account for a substantial portion of cardiovascular healthcare expenditures, with repeated hospitalizations driving costs higher [41]. In Syria, where the healthcare system is already strained, the high frequency of readmissions among elderly AF patients further overwhelms limited hospital resources, reducing the capacity for effective long‐term management. Addressing these challenges requires targeted interventions, including structured discharge planning, enhanced outpatient care, and better risk stratification to identify high‐risk individuals who may benefit from closer follow‐up [42].

## 5 Limitations

Data collection was confined to a single tertiary care center in Latakia. This city was comparatively less impacted by the Syrian conflict than the other eastern and northern regions of Syria. Therefore, our findings may not apply to other areas or centers due to the considerable variation in the quality and availability of hospital resources and personnel. Moreover, our analysis only included data routinely documented in medical records and the number of patients visiting the hospital. Consequently, there may be additional factors influencing mortality that have not been identified. This study did not assess treatments during the initial admission or discharge process, which might have affected the results. Readmissions to centers outside those studied were not included, which may result in underestimating the readmission rate.

## 6 Conclusion

This study highlights the substantial burden of 1‐year readmissions among AF patients in conflict‐stricken Syria, revealing a readmission rate of 64%, markedly higher than in developed countries. Older age significantly increases the risk of both all‐cause and cardiovascular readmissions, emphasizing the vulnerability of elderly patients. AF recurrence was the primary reason for readmissions, reflecting ongoing healthcare challenges due to conflict‐related resource limitations. Strengthening healthcare infrastructure, improving access to care, and addressing modifiable risk factors are urgently needed to alleviate this burden and enhance patient outcomes in similar settings.

## Author Contributions

IA designed the study, analyzed the data, and wrote the first draft of the manuscript. AA and AA managed data collection. MZ, GAN and RS reviewed and edited the manuscript.

## Ethics Statement

The study was conducted as part of an audit approved by the hospital board and involved prospective analysis of retrospectively collected anonymised data. Therefore, the ethical committee of Tishreen's University Hospital waived the need for consent.

## Conflicts of Interest

The authors declare no conflicts of interest.

## Data Availability

The data that support the findings of this study are available from the corresponding author upon reasonable request.
